# Maternal and Perinatal Outcome in Women with Systemic Lupus Erythematosus: A Retrospective Bicenter Cohort Study

**DOI:** 10.1155/2017/8245879

**Published:** 2017-09-28

**Authors:** Sylvia J. Kroese, Carolien N. H. Abheiden, Birgit S. Blomjous, Jacob M. van Laar, Ronald W. H. M. Derksen, Irene E. M. Bultink, Alexandre E. Voskuyl, A. Titia Lely, Marjon A. de Boer, Johanna I. P. de Vries, Ruth D. E. Fritsch-Stork

**Affiliations:** ^1^Department of Rheumatology & Clinical Immunology, University Medical Center Utrecht, P.O. Box 85500, 3508 GA Utrecht, Netherlands; ^2^Department of Obstetrics and Gynecology, VU University Medical Center, P.O. Box 7057, 1007 MB Amsterdam, Netherlands; ^3^Department of Rheumatology, Amsterdam Rheumatology and Immunology Center, VU University Medical Center, P.O. Box 7057, 1007 MB Amsterdam, Netherlands; ^4^Department of Obstetrics and Gynecology, University Medical Center Utrecht, P.O. Box 85500, 3508 GA Utrecht, Netherlands; ^5^1st Medical Department and Ludwig Boltzmann Institute of Osteology at the Hanusch Hospital of WGKK, Vienna, Austria; ^6^AUVA Trauma Centre Meidling, Hanusch Hospital, Vienna, Austria; ^7^Sigmund Freud University, Vienna, Austria

## Abstract

**Objective:**

To investigate disease activity around and during pregnancy and pregnancy outcome in women with systemic lupus erythematosus (SLE) considering antiphospholipid antibody status. Moreover, differences between first and consecutive pregnancies were examined.

**Methods:**

Pregnancies > 16 weeks gestation of SLE patients receiving joint care from rheumatologists and gynecologists in two tertiary centers in the Netherlands between 2000 and 2015 were included. Disease activity, flare rate, and pregnancy outcomes and complications were assessed.

**Results:**

Ninety-six women (84% Caucasian) with 144 pregnancies were included. The median SLE(P)DAI score was 2 before, during, and after pregnancy. Flare rates were 6.3%, 20.1%, and 15.3%, respectively. Severe hypertensive disorder of pregnancy, intrauterine fetal death, preterm birth, and small-for-gestational age infants occurred in 18.1%, 4.1%, 32.7%, and 14.8%, respectively. Complication rates were similar in the first and consecutive pregnancies. Half of the women did not experience any pregnancy complication whereas 42.7% developed a complication during all pregnancies. Mean number of pregnancies was 2.4 and live births 1.7.

**Conclusion:**

In this SLE population with low disease activity, pregnancy complications were present irrespective of antiphospholipid antibody status. Furthermore, there were no differences in complication rates between the first and consecutive pregnancies as seen in healthy mothers. This information is useful for patient counseling.

## 1. Introduction

Systemic lupus erythematosus (SLE) is a systemic autoimmune disease that often affects women during their childbearing age [1]. It is well known that women with SLE may experience an increase in disease activity during pregnancy [2–4]. Moreover, women with SLE have a higher risk of experiencing pregnancy complications like hypertensive disorders (HD) of pregnancy (pregnancy-induced hypertension (PIH); preeclampsia; eclampsia; and hemolysis, elevated liver enzymes, and low platelet count (HELLP) syndrome), preterm birth, intrauterine fetal death (IUFD), and small-for-gestational age (SGA) infants compared to the general population [4–7].

Several risk factors for pregnancy complications in women with SLE have been reported, amongst them are the presence of antiphospholipid antibodies (aPL) or antiphospholipid syndrome (APS), (prior) lupus nephritis, and active disease at conception [8–12]. Therefore, low disease activity for at least six months is recommended to lower the risk for SLE flares and maternal and perinatal complications [13–16]. In order to achieve this, preconceptional counseling and close collaboration between gynecologist and rheumatologist are recommended [10, 13, 17]. Evaluation of risk factors (e.g., smoking, hypertension, overweight, and family history) and optimization of timing of pregnancy are goals of preconceptional counseling. Moreover, the use of pregnancy compatible medication, amongst others azathioprine and hydroxychloroquine (HCQ), is evaluated in order to prevent flares and maternal and perinatal complications [18].

Over the last decades, an improvement in pregnancy outcomes in SLE patients has been reported [19]. A recent large North-American multicenter study investigated one pregnancy per woman with SLE (*n* = 385), excluding patients with comorbidity such as diabetes or impaired renal function and patients using medium or high-dose glucocorticosteroids. The results of this study demonstrated that 80% of the neonates was born alive after a gestation period >36 weeks, not including miscarriages [20].

In the present study, pregnancies of women with SLE over a 16-year period, irrespective of comorbidity and medication use, are described.

In the general population, HD and PIH occur most commonly in the first pregnancies [21]. This has not been examined yet in a population with SLE, where several factors (e.g., underlying immune activation, impaired renal function, or APS) might be associated with a higher incidence of HD and other pregnancy complications also in consecutive pregnancies.

The aim of the present study is to examine three topics, taking the antiphospholipid antibody status into account:

1. SLE disease activity before, during, and after pregnancy per pregnancy
2. Maternal and perinatal complications occurring in the first and consecutive pregnancies and during the reproductive period
3. Total number of live births per patient

The results of this study will provide relevant information for health care professionals who are involved in the treatment and preconceptional counseling of these patients and their partners.

## 2. Patients and Methods

This cohort study involved two tertiary centers in the Netherlands: the University Medical Center Utrecht and the VU University Medical Center in Amsterdam. To identify pregnancies in women with SLE, a search was performed in both obstetric and rheumatology databases. Data were retrieved from medical files and collected in both centers using the same case report form. The Institutional Review Boards of both university hospitals concluded that official approval from a medical ethical committee was not needed due to the observational character of this study.

### 2.1. Participants

Inclusion criterion was diagnosis of SLE according to the American College of Rheumatology (ACR) revised criteria [22], diagnosed before the start of the first recorded study pregnancy. To contain uniformity in the classification of SLE, only the ACR revised criteria were used for all pregnancies, even though in 2012 new SLE classification criteria were published [23]. Moreover, only patients with both obstetric and rheumatology check-ups during pregnancy in one of the two participating centers were included. All ongoing pregnancies (>16 weeks of gestation) between the years 2000 and 2015 were included. No exclusion criteria were formulated.

Antiphospholipid antibody status was recorded in all patients. Patients were divided into SLE without aPL, SLE with aPL, or SLE with APS. Presence of aPL was defined as two positive measurements of either IgG or IgM anticardiolipin antibodies or lupus anticoagulant, measured at least six weeks (before 2006) or 12 weeks (after 2006) apart and, when applicable, not during pregnancy or within ten weeks thereafter [24, 25]. In 29.9% of the pregnancies, presence of beta-2-glycoprotein antibodies was measured as well. Samples were considered positive for anticardiolipin antibodies or beta-2-glycoprotein antibodies when either above 40 GPL, 40 MPL, or above the 99th percentile. APS was diagnosed according to the Sapporo criteria [24].

### 2.2. Outcomes

Baseline characteristics included information about aPL status, demographic background, age, body mass index (BMI), and general and obstetric history. The obstetric history included miscarriages (<16 weeks gestation), severe HD (preeclampsia, eclampsia, and HELLP syndrome), IUFD, preterm birth (<37 weeks), and SGA infants (birth weight < p10).

Disease activity was assessed in retrospect using the Systemic Lupus Erythematosus Disease Activity Index (SLEDAI) within 6 months before pregnancy and within 6 months postpartum [26]. In each trimester of pregnancy, disease activity was assessed using SLEPDAI (SLEDAI adjusted for pregnancy) [27]. Flares were defined according to the Safety of Estrogens in Lupus Erythematosus National Assessment (SELENA) SLEDAI definitions [28]. Clinical manifestations of SLE (according to the ACR revised criteria [22]) and (changes in) dosages of medication were registered. Anticoagulation therapy during pregnancy of this population has been published [29].

The following maternal and perinatal complications were scored: mild HD (PIH), severe HD, IUFD, preterm birth (both <36 and <37 weeks gestation), SGA infants, and occurrence of neonatal lupus: either cutaneous lupus or congenital heart block. Occurrence of HD (and thereby distinction with nephritis) was scored by one of the gynecologists (ATL and JIPdV).

In the first analysis, maternal and perinatal complications were described for all pregnancies meeting the inclusion criteria; secondly, a comparison between the first and consecutive pregnancies meeting the inclusion criteria was undertaken. In the third analysis, all complications per included patient during the studied reproductive period were examined. The latter was defined as the study pregnancies during the 16-year period and prior obstetric history.

Data are presented per total SLE population and per any of the three subdivisions: aPL absent, aPL present but not fulfilling APS criteria, and aPL present plus fulfillment of APS criteria.

Total number of pregnancies, total number of live births, and miscarriage rate per patient were examined during the studied reproductive period.

### 2.3. Statistics

Baseline characteristics were examined per antiphospholipid group using Fisher's exact test or chi-square test for dichotomous variables and independent samples median test or one-way ANOVA for continuous variables.

Differences in disease activity and start or increase of prednisone, azathioprine, and HCQ dose between the three antiphospholipid groups were tested using independent samples median test for continuous variables or Fisher's exact test for dichotomous variables. Total number of flares during pregnancy compared with the total number of flares postpartum was examined using a Fisher's exact test.

Differences in incidence rates of maternal and perinatal complications between the three aPL subdivisions were investigated using generalized estimating equations, for which an exchangeable correlation structure was chosen. This analysis corrects for patient dependency since some women in our cohort were included with multiple pregnancies. All outcomes were corrected for smoking, body mass index (BMI) >25 kg/m^2^, and prednisone use.

Differences in maternal and perinatal complication rates between the first and consecutive pregnancies were examined using a chi-square test or Fisher's exact test.

Maternal and perinatal complication rates and numbers of live births in the studied reproductive period were studied using descriptive statistics.

Statistical analysis was performed using SPSS for Windows (version 22, SPSS Inc., Chicago, IL, USA). A two-sided *p* value inferior to 0.05 was considered to be statistically significant.

## 3. Results

### 3.1. Baseline Characteristics

In total, 96 patients with 144 pregnancies met the inclusion criteria. Distribution of the parity was 70 nulliparous women and 18 primiparous women, 7 women were para 2, and 1 woman was para 4 at the first included study pregnancy. Baseline characteristics are presented in Table 1. In the group of 10 patients with SLE and APS, nine had a history of thrombotic APS and four had a history of obstetric APS. LAC status was positive in 28.6% of the patients with SLE and aPL and positive in 84.6% of the patient with SLE and APS. Of the non-Caucasian patients, eight were black and seven women were Asian. Thirty-three percent of all patients had a BMI above 25 (kg/m^2^). None of the patients had a platelet count below 100 × 10^9^/L at the start of the pregnancy. The only significant difference in baseline characteristics between groups was the inherent significantly increased history of thrombosis in APS-diagnosed women.

### 3.2. SLE Manifestations and Disease Activity before, during, and after Pregnancy

#### 3.2.1. ACR Criteria

Most frequently found ACR criteria contributing to the diagnosis of SLE before pregnancy were arthritis, renal involvement, positive ANA, and hematological (leuco-, lymphocyto-, or thrombocytopenia, hemolytic anemia) and/or immunological (anti-dsDNA, anti-Smith, or anticardiolipin antibodies or LAC) anomalies. There were no differences in prevalence of the ACR criteria between both centers (data not shown). Arthritis was significantly less often found in the SLE + APS group (*p* = 0.01); the other ACR criteria did not show statistically significant differences between the groups.

#### 3.2.2. Disease Activity and Flares

Results of disease activity measurements according to the SLE(P)DAI criteria before and during pregnancy and postpartum are presented in Table 2. Only the SLEPDAI score during the 3rd trimester was significantly lower in the SLE-aPL group compared to the other groups (*p* < 0.01). In 30.6% of pregnancies, a flare occurred during the pregnancy period or within six months before or after the pregnancy. Flares were mostly characterized by decreased complement levels, hematuria, proteinuria, rash, or arthritis. Of nine pregnancies in which a mild flare occurred within six months before pregnancy, five experienced consecutive flares either during pregnancy (*n* = 1), postpartum (*n* = 3), or both (*n* = 1). Severe flares occurred three times during pregnancy and twice postpartum. These severe flares were characterized by (amongst others) nephritis, pleuritis, and rash. One patient had both mild flares (during pregnancy) as well as a severe flare (postpartum).

Flare rates postpartum were lower than during pregnancy (15.3 versus 20.1% resp., *p* < 0.01). Out of 22 pregnancies in which a flare occurred postpartum, 14 also had a flare before or during pregnancy (63.6%, *p* < 0.01). In total, 61 flares occurred in 44 pregnancies.

#### 3.2.3. Medication Use

Treatment with prednisone, azathioprine, or HCQ was started or dosages were increased during pregnancy in 17%, 4%, and 3% of the pregnancies, respectively (Table 2). Frequencies of initiation or dose increase of prednisone or azathioprine during pregnancy did not differ per antiphospholipid group (*p* = 0.77 and *p* = 0.72, resp.). Initiation or dose increase of HCQ during pregnancy was more frequent in patients with SLE + APS compared to the other two groups (*p* < 0.01).

In 54 pregnancies, HCQ was used. Comparing the treatment before and after 2008, the use of HCQ during pregnancy increased: 16% received HCQ before 2008 and 58% after 2008 (*p* < 0.01). Flare rate during pregnancy (*p* = 0.09), occurrence of severe HD (*p* = 0.31), IUFD (*p* = 0.20), or preterm birth < 37 weeks (*p* = 0.75) did not differ before and after 2008.

### 3.3. Maternal and Perinatal Complications

Maternal and perinatal complications of all study pregnancies are presented in Table 3. In total, there were three twin pregnancies.

#### 3.3.1. Maternal Complications

HELLP syndrome was found statistically significantly more often in the SLE + APS group compared to the other groups (*p* < 0.01). A placental abruption occurred in one pregnancy in a patient with SLE without aPL. In 36.8% of all pregnancies, a caesarian section was conducted, which was not different between the groups (*p* = 1.00).

#### 3.3.2. Perinatal Complications

Of all preterm births (<37 weeks), 44.2% occurred spontaneously, and in the others, labour was induced. Main indications for preterm induction of labour (<37 weeks) were HD (54.1%) and IUFD (12.5%). Of all pregnancies, 32.7% ended before 37 weeks and 24.3% before 36 weeks. Of all live-born infants, 55.3% were admitted to the medium care or neonatal intensive care unit. No neonatal deaths occurred. Of the two infants with neonatal lupus, one had a congenital heart block and the other cutaneous lupus. Congenital anomalies occurred in nine pregnancies of which the overwhelming majority [8] occurred within the SLE-aPL group, albeit not statistically significant (*p* = 0.06). Anomalies included trisomy 21 with Fallot's tetralogy, premature retinopathy, omphalocele, macrosomia, clubfoot, anus atresia, hip dysplasia, congenital (bilateral) glaucoma (2 children), and muscular ventricular septal defect. The congenital glaucoma occurred in siblings.

#### 3.3.3. Pregnancy Complications in the First and Consecutive Pregnancies

**Figure 1:**
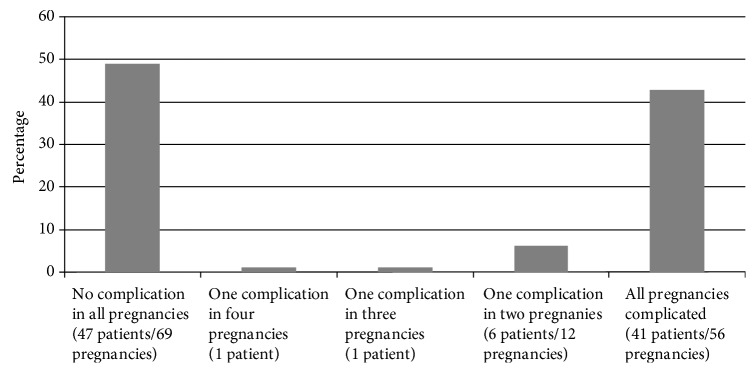
Percentage of pregnancy complications during the studied reproductive period. Complications included presence of any of the following: severe hypertensive disorders of pregnancy (including preeclampsia, eclampsia, and HELLP syndrome), placental abruption, preterm birth < 37 weeks, intrauterine fetal death, or small-for-gestational age infant.

The incidence of pregnancy complications did not differ between the first (*n* = 70) and consecutive (*n* = 74) pregnancies, with severe HD occurring in both first and consecutive pregnancies in 18.6% and 17.6%, respectively (*p* = 0.88), preterm birth < 37 weeks in 36.6% and 28.9%, respectively (*p* = 0.32), IUFD in 4.2% and 3.9%, respectively (*p* = 1.00) and SGA in 14.7% and 14.9%, respectively (*p* = 0.98). The rates of maternal and perinatal pregnancy complications for the studied reproductive period per patient are presented in Figure 1. Complications mostly occurred either in none of the pregnancies in the reproductive study period or in all studied pregnancies.

### 3.4. Live Births

The mean number of live births was 1.7 ± 0.8 standard deviation (SD) per patient during the studied reproductive period. The mean number of pregnancies per patient was 2.4 ± 1.4 SD (including miscarriages), and miscarriage rate was 14% with a mean of 0.33 ± 0.7 SD per woman during the studied reproductive period.

## 4. Discussion

In this study, we investigated the disease activity and maternal and perinatal complications of ongoing pregnancies (>16 weeks) in patients with SLE in the Netherlands in a real-life setting. Low disease activity was found before, during, and after pregnancy. Still, the incidence of maternal as well as perinatal complications was higher (especially preterm birth rate), compared to the general population, regardless of the overall low disease activity [30, 31]. The prevalences of severe HD, preterm birth, IUFD, and SGA infants were similar irrespective of antiphospholipid antibody status. One exception was HELLP syndrome, occurring more frequently in patients with SLE and APS. However, comparison between groups is difficult since the number of pregnancies within the SLE + aPL and the SLE + APS group were only 14 and 13, respectively. We also observed a similar complication rate in the first and consecutive pregnancies in contrast to the general population and a number of live births per women of 1.7. Both these findings are discussed later on.

In our patient population, only 6.3% experienced a mild flare before pregnancy, and no severe flares occurred. This is a reflection of planned parenthood facilitated by the close collaboration between rheumatologists and gynecologists in our centers. The (on average) low disease activity before pregnancy likely contributed to the low flare rate during pregnancy of around 20%, which is comparable with other studies reporting incidence rates between 10 and 33% [32–34]. The incidence of postpartum flares (<6 months) in our cohort was amounting to 15%. Importantly, patients with a flare during pregnancy were at greatest risk to develop a flare postpartum, and vice versa: 63.6% of flare postpartum occurred in patients with a flare before or during pregnancy. This finding calls for even more vigilance in the postpartum period especially in patients with increased disease activity during pregnancy.

The increase in use of HCQ after 2008 in the present study was neither associated with lower median disease activity scores during pregnancy nor associated with a reduced incidence of pregnancy complications. This finding is partly in line with the results of a recent retrospective study which demonstrated no difference in flare rates or maternal pregnancy complications such as severe HD and IUFD between patients treated with and without HCQ [32]. On the other hand, in the latter study, a reduction of mild HD (PIH) and preterm birth < 37 weeks was seen. Moreover, a prospective cohort study where HCQ was used in a similar number of pregnancies compared to the present study, and a small RCT suggested lower disease activity during pregnancy when HCQ was used [12, 18, 35]. This might be partly due to a different study population since we had an overwhelming majority of Caucasian women (84%) as opposed to Clowse et al. and Levy et al. (approximately 50–60%) [18, 35]. As SLE manifestations are known to be more severe in non-Caucasian women, the effect of HCQ may be greater in these patients. Also, adherence to medication could have played a role; we did not measure drug metabolites in the blood.

Maternal pregnancy complications occurred more often in our patients compared to those reported in the general population, including mild and severe forms of HD and preterm birth [30, 31]. In the general population, HD affect about 5–10% of all pregnancies and preterm birth occurs in less than 10% of all pregnancies in developed countries [30, 31]. The observed rate of 18.1% of HD in the present study is in line with other studies performed within patients with SLE [6, 36]. The percentage of patients who developed HELLP syndrome is low in our study with a significantly higher occurrence within the SLE + APS group compared to the other groups. Recently, Moroni et al. described maternal outcomes of prospectively followed pregnancies in women with a history of lupus nephritis and found a significant association between anti-beta-2 IgM antibody levels and preeclampsia/HELLP (*p* = 0.048) [11]. In our cohort, we only found an association with APS and HELLP. However, considering the low numbers of HELLP in the paper by Moroni et al. (2 out of 71 pregnancies = 2.6%) and our study (7 out of 144 pregnancies = 4.9%), we do not venture to interpret these findings.

We described that all patients with SLE + APS were treated with low-molecular-weight heparin during pregnancy [29]. This is in agreement with the perceived increased occurrence of HELLP syndrome in patients with primary APS compared to mere aPL positivity in the literature [37, 38]. These findings suggest an important but not exclusive role for antiphospholipid antibodies in the development of HELLP syndrome.

The preterm birth rate was lower in the recent PROMISSE study, a prospective cohort study, compared to our study: 9% versus 24.3% < 36 weeks gestation, respectively [20]. This discrepancy might be explained by differences in the design of both studies and the definition of preterm (<37 weeks in our cohort versus <36 weeks in the PROMISSE study). In the PROMISSE study, patients with important comorbidity, for example, patients with diabetes mellitus or urinary protein–creatinine ratio greater than 1000 mg/g and patients using medium or high dosages of glucocorticoids were excluded. The preterm birth rate < 37 weeks found in our study is in line with the results of other studies [12, 39, 40].

We did not find a relevant increase in congenital abnormalities in our study. Congenital glaucoma, a rare congenital deformity with a prevalence of approximately 1 : 10,000 in Western population, occurred twice in our cohort, both children from the same mother suggesting a non-SLE-associated genetic influence, as was shown by Gencik et al. [41, 42].

To our knowledge, we compared for the first time the incidence of complications during the first and consecutive pregnancies in an SLE cohort. Shand et al. reported consecutive pregnancy outcomes within SLE patients using birth records without taking SLE disease characteristics into account [43]. We demonstrated that incidences of HD, preterm birth < 37 weeks, IUFD, and SGA are similar for consecutive pregnancies compared to the first pregnancy. This finding is not in line with observations in the general population, where nulliparity has been demonstrated as a risk factor for HD and multiparity reduces this risk, probably due to improvement of maternal-fetal immune adaptation in subsequent pregnancies [44]. We postulate that the maternal-fetal immune adaptation is different in patient with SLE. Patients with SLE should be informed about this finding in the preconceptional counseling.

Moreover, we examined pregnancy complications during the studied reproductive period. Remarkably, almost half of the patients did not develop any severe complication during all of their pregnancies and more than 50% developed at least one severe complication during any of their pregnancies. Although our finding may have been hampered by recall bias, it is a useful additional information in the preconceptional counseling.

The mean number of pregnancies per woman in our study was 2.4 and resulted in a mean number of live births of 1.7. This is similar to results of a case-control study in the late nineties with a mean number of pregnancies of 2.3 and mean number of live births of 1.8 [45]. Limitation of that case-control study is that the severity of SLE was not described. A review examining pregnancy loss (not further defined) showed a decrease of pregnancy loss between 1960 and 2000 [19]. The results of our study implicate that no further improvement in the number of pregnancies and number of live births has occurred over the last 15 years, although it is unknown if our population (with a history of nephritis and thrombosis in 39.6% and 16.0% of the pregnancies, resp.) is comparable with the population of Hardy et al. considering the information given in the publication [45].

The strength of the present study is that we did not use exclusion criteria with respect to disease activity, comorbidity, medication use, and twin pregnancies which enables us to present pregnancy outcomes of a complete SLE population reflecting real-life setting. Furthermore, by including all pregnancies per woman during a 16-year period of time, we were able to examine pregnancy complications between the first and consecutive pregnancies which, to our knowledge, have not been described before. A weakness of our study is that the majority of the population consisted of SLE patients without aPL which limits optimal comparison of women with aPL or APS.

## 5. Conclusions

In our cohort of 96 patients and 144 pregnancies, we made several observations: the incidence of maternal and perinatal complications is high compared to the reported rates in the general population, irrespective of antiphospholipid antibody status, despite low disease activity before, during, and after pregnancy. Furthermore, we compared for the first time disease activity and pregnancy outcomes in the first and consecutive pregnancies in patients with SLE and found that the incidence rates of HD, preterm birth < 37 weeks, IUFD, and SGA infants did not decrease in consecutive pregnancies, in contrast to the general population. Additionally, almost half of the women did not experience a severe complication including severe HD, placental abruption, preterm birth (<37 weeks), IUFD, and SGA infant in any pregnancy during their studied reproductive period (obstetric history and study). These observations could be of additional value in future counseling of SLE patients.

## Figures and Tables

**Table 1:** Baseline characteristics per study pregnancy.

Pregnancies (*n*)	Total 144	SLE-aPL 117	SLE + aPL 14	SLE + APS 13	*p* value
Number of women	96	77	9	10	NA
Study pregnancies per woman	1 [1-2]	1 [1-2]	1 [1-2]	1 [1–1.5]	0.71
Non-Caucasian^#^	15/91 (16.5)	13/74 (17.6)	2/8 (25.0)	0/9 (0)	0.36
Age (years)	31.9 ± 4.4	32.1 ± 4.4	29.7 ± 4.0	32.5 ± 4.7	0.16
BMI (kg/m^2^)	23.7 ± 4.4	23.2 ± 3.5	25.2 ± 3.6	25.9 ± 9.0	0.05
Smoking	12/139 (8.6)	10/113 (8.8)	0/14 (0)	2/12 (16.7)	0.27
*General history*					
Chronic hypertension	20/142 (14.1)	16/117 (13.7)	2/13 (15.4)	2/12 (16.7)	0.81
Diabetes	5/143 (3.5)	4/117 (3.4)	0/14 (0)	1/12 (8.3)	0.42
History of thrombosis^∗^	23/144 (16.0)	14/117 (12.0)	0/0 (0)	9/13 (69.2)	<0.01
Serum creatinine level < 6 months before pregnancy (*μ*mol/L)	67.2 ± 11.4	67.6 ± 10.9	69.0 ± 13.4	62.6 ± 13.2	0.31
*SLE-specific history*					
SLE duration before start pregnancy (years)	7.8 ± 4.9	7.7 ± 5.0	8.7 ± 4.2	7.6 ± 4.6	0.76
History of nephritis^^^	57/144 (39.6)	45/117 (38.5)	7/14 (50.0)	5/13 (38.5)	0.70
SS-A and/or SS-B positive	70/138 (50.7)	61/111 (55.0)	6/14 (42.9)	3/13 (23.1)	0.08
*Medication use at start pregnancy*					
Hydroxychloroquine	69/135 (51.1)	54/109 (49.5)	6/13 (46.2)	9/13 (69.2)	0.38
Azathioprine	39/140 (27.6)	35/114 (30.7)	3/13 (23.1)	1/13 (7.7)	0.21
Prednisone	74/140 (52.9)	63/114 (55.3)	7/13 (53.8)	4/13 (30.8)	0.25
*Obstetric history*					
Miscarriages^∞^	32/94 (34.0)	26/78 (33.3)	2/8 (25.0)	4/8 (50.0)	0.68
Severe HD	19/63 (30.2)	16/51 (31.4)	2/7 (28.6)	1/5 (20.0)	1.00
IUFD	13/91 (14.3)	13/76 (17.1)	0/8 (0)	0/7 (0)	0.33
Preterm birth (<37 weeks)	24/76 (31.6)	21/64 (32.8)	2/7 (28.6)	1/5 (20.0)	1.00
SGA infant	17/69 (24.6)	16/57 (28.1)	0/14 (0)	1/5 (20.0)	0.34

Data depicted as numbers (%), mean ± standard deviation, or median [interquartile range]. ^#^This item is depicted per woman, not per pregnancy, ^∗^either arterial or venous, ^^^biopsy proven, ^∞^<16 weeks gestation. SLE: systemic lupus erythematosus; aPL: antiphospholipid antibodies; APS: antiphospholipid syndrome; NA: not applicable; BMI: body-mass index; severe HD: hypertensive disorders of pregnancy including preeclampsia, eclampsia, and HELLP syndrome; IUFD: intrauterine fetal death; SGA: small-for-gestational age (birth weight < p10).

**Table 2:** Disease activity before, during, and after pregnancy and medication alterations.

	Total (*n* = 144)	SLE-aPL (*n* = 117)	SLE + aPL (*n* = 14)	SLE + APS (*n* = 13)	*p* value
SLEDAI < 6 months before pregnancy	2 [0–4]	2 [0–4]	2 [0–4]	3 [2–4]	0.22
SLEPDAI 1st trimester	2 [0–2]	2 [0–2]	2 [0–2]	2 [0.5–2]	0.41
SLEPDAI 2nd trimester	2 [0–2]	2 [0–2]	2 [1–3]	2 [0.5–2]	0.74
SLEPDAI 3rd trimester	2 [0–2]	0 [0–2]	2 [2–4.5]	2 [0.5–5.5]	<0.01
SLEDAI < 6 months postpartum	2 [0–4]	2 [0–4]	2 [0–5]	4 [2–5.5]	0.27
Any flare before, during pregnancy, or postpartum	44/144 (30.6)	35/117 (29.9)	5/14 (35.7)	4/13 (30.8)	0.94
Severe flare before, during pregnancy, or postpartum	5/144 (3.5)	5/117 (4.3)	0/14 (0)	0/13 (0)	1.00
Mild/moderate flare before, during pregnancy, or postpartum^∗^	40/144 (27.8)	31/117 (26.5)	5/14 (35.7)	4/13 (30.8)	0.73
<6 months before pregnancy	9/144 (6.3)	8/117 (6.8)	0/14 (0)	1/13 (7.7)	0.67
1st trimester	6/144 (4.2)	5/117 (4.3)	1/14 (7.1)	0/13 (0)	0.72
2nd trimester	14/144 (9.7)	9/117 (7.7)	3/14 (21.4)	2/13 (15.4)	0.11
3rd trimester	7/144 (4.9)	6/117 (5.1)	1/14 (7.1)	0/13 (0)	0.77
<6 months postpartum	20/144 (13.9)	17/117 (14.5)	2/14 (14.3)	1/13 (7.7)	0.91
Medication started or dosage increased during pregnancy					
Prednisone	25/144 (17)	22/117 (19)	2/14 (14)	1/13 (8)	0.77
Prednisone	25/144 (17)	22/117 (19)	2/14 (14)	1/13 (8)	0.77
Azathioprine	6/144 (4)	5/117 (4)	1/14 (7)	0/13 (0)	0.72
Hydroxychloroquine	4/144 (3)	1/117 (1)	0/14 (0)	3/13 (23)	<0.01

Data depicted as median [interquartile range] or numbers (%). ^∗^A woman can flare multiple times before or during pregnancy or postpartum. SLE: systemic lupus erythematosus; aPL: antiphospholipid antibodies; APS: antiphospholipid syndrome; SLEDAI: SLE disease activity index; SLEPDAI: SLE disease activity index adjusted for pregnancy.

**Table 3:** Maternal and perinatal pregnancy complications in all study pregnancies.

	Total	SLE-aPL	SLE + aPL	SLE + APS	*p* value
*Maternal complications*	*N* = 144	*N* = 117	*N* = 14	*N* = 13	
Mild HD	21/144 (14.6)	18/117 (15.4)	1/14 (7.1)	2/13 (15.4)	0.82
Severe HD	26/144 (18.1)	19/117 (16.2)	3/14 (21.4)	4/13 (30.8)	0.32
Preeclampsia	24/140 (17.1)	18/113 (15.9)	3/14 (21.4)	3/13 (23.1)	0.82
Onset preeclampsia < 34 weeks	8/24 (33.3)	7/18 (38.9)	1/3 (33.3)	0 (0)	1.00
Eclampsia	1/139 (0.7)	1/112 (0.9)	0/14 (0)	0/13 (0)	1.00
HELLP syndrome	7/144 (4.9)	3/117 (2.6)	1/14 (7.1)	3/13 (23.1)	<0.01
*Perinatal complications* ^∗^	*N* = 147	*N* = 119	*N* = 15	*N* = 13	
IUFD	6/147 (4.1)	6/119 (5.0)	0/15 (0)	0/13 (0)	1.00
Preterm birth (<37 weeks)	48/147 (32.7)	40/119 (33.6)	4/15 (26.7)	4/13 (30.8)	0.95
SGA infant	21/142 (14.8)	18/115 (15.7)	2/15 (13.3)	1/12 (8.3)	0.77
Neonatal lupus	2/147 (1.4)	2/119 (1.7)	0/15 (0)	0/13 (0)	1.00

Data depicted as numbers (%). ^∗^There were three twin pregnancies. SLE: systemic lupus erythematosus; aPL: antiphospholipid antibodies; APS: antiphospholipid syndrome; mild HD: hypertensive disorders of pregnancy including pregnancy induced hypertension; severe HD: hypertensive disorders of pregnancy including preeclampsia, eclampsia, and HELLP (hemolysis, elevated liver enzyme, and low platelet count syndrome); IUFD: intrauterine fetal death; SGA: small-for-gestational age (birth weight < p10).
