# Corrigendum to “Maternal and Perinatal Outcome in Women with Systemic Lupus Erythematosus: A Retrospective Bicenter Cohort Study”

**DOI:** 10.1155/2022/9828675

**Published:** 2022-04-26

**Authors:** Sylvia J. Kroese, Carolien N. H. Abheiden, Birgit S. Blomjous, Jacob M. van Laar, Ronald W. H. M. Derksen, Irene E. M. Bultink, Alexandre E. Voskuyl, A. Titia Lely, Marjon A. de Boer, Johanna I. P. de Vries, Ruth D. E. Fritsch-Stork

**Affiliations:** ^1^Department of Rheumatology & Clinical Immunology, University Medical Center Utrecht, P.O. Box 85500, 3508 GA Utrecht, Netherlands; ^2^Department of Obstetrics and Gynecology, VU University Medical Center, P.O. Box 7057, 1007 MB Amsterdam, Netherlands; ^3^Department of Rheumatology, Amsterdam Rheumatology and Immunology Center, VU University Medical Center, P.O. Box 7057, 1007 MB Amsterdam, Netherlands; ^4^Department of Obstetrics and Gynecology, University Medical Center Utrecht, P.O. Box 85500, 3508 GA Utrecht, Netherlands; ^5^1st Medical Department and Ludwig Boltzmann Institute of Osteology at the Hanusch Hospital of WGKK, Vienna, Austria; ^6^AUVA Trauma Centre Meidling, Hanusch Hospital, Vienna, Austria; ^7^Sigmund Freud University, Vienna, Austria

In the article titled “Maternal and Perinatal Outcome in Women with Systemic Lupus Erythematosus: A Retrospective Bicenter Cohort Study” [1], the authors identified some discrepancies in the data following its publication.

There are very small changes in incidence of HELLP syndrome (*N* = 5 instead of *N* = 7), preeclampsia (*N* = 22 instead of 24), and mild hypertensive disorder (*N* = 19 instead of 21), which showed no significant difference in the preeclampsia group. The incidence of preeclampsia <34 weeks, eclampsia, and HELLP syndrome were too low to conduct the generalized estimating equations (GEE) analysis; therefore, a new *p* value cannot be produced. Due to the very small differences, we believe that our conclusions are not changed. As already described on page 6 of the article: “In our cohort, we only found an association with APS and HELLP. However, considering the low numbers of HELLP in the paper by Moroni et al. (2 out of 71 pregnancies = 2.6%) and our study (7 out of 144 pregnancies = 4.9%), we do not venture to interpret these findings.”

The corrected Table 3 is shown below.

Additionally, in Section 3.3.2, the sentence “Of all preterm births (<37 weeks), 44.2% occurred spontaneously, and in the others, labour was induced. Main indications for preterm induction of labour (<37 weeks) were HD (54.1%) and IUFD (12.5%).” should be corrected to “Of all preterm births (<37 weeks), 61.9% occurred spontaneously. Main indications for preterm induction of labor (<37 weeks) were HD (30.8%) and IUGR (23.1%). IUFD as reason for preterm induction of labor occurred once (7.7%).”

Also, the email address of the corresponding author should be changed to “s.kroese@hagaziekenhuis.nl.”

The authors confirm that this does not affect the results and conclusions of the article, and the editorial board agrees to the publication of a corrigendum.

## Figures and Tables

**Table 3:** Maternal and perinatal pregnancy complications in all study pregnancies.

	Total	SLE-aPL	SLE + aPL	SLE + APS	*p* value
*Maternal complications*	*N* = 144	*N* = 117	*N* = 14	*N* = 13	
Mild HD	19/144 (13.2)	16/117 (13.7)	1/14 (7.1)	2/13 (15.4)	ns
Severe HD	24/144 (16.7)	18/117 (15.4)	2/14 (14.3)	4/13 (30.8)	ns
Preeclampsia	22/141 (15.6)	17/114 (14.9)	2/14 (14.3)	3/13 (23.1)	ns
Onset preeclampsia <34 weeks	8/22 (36.4)	7/17 (41.2)	1/3 (33.3)	0 (0)	—
Eclampsia	1/140 (0.7)	1/113 (0.9)	0/14 (0)	0/13 (0)	—
HELLP syndrome	5/144 (3.5)	3/117 (2.6)	0/14 (0)	2/13 (15.4)	—
*Perinatal complications*	*n* = 147	*n* = 119	*n* = 15	*n* = 13	
IUFD	6/147 (4.1)	6/119	0/15	0/13	ns
Preterm birth	48/147 (32.7)	40/119 (33.6)	4/15 (26.7)	4/13 (30.8)	ns
SGA infant	21/142 (14.8)	18/115 (15.7)	2/15 (13.3)	1/12 (8.3)	ns
Neonatal lupus	2/147 (1.4)	2/119 (1.7)	0/15 (0)	0/13 (0)	ns
